# Clinical and imaging manifestations of primary cardiac angiosarcoma

**DOI:** 10.1186/s12880-019-0318-4

**Published:** 2019-02-14

**Authors:** Jin-Fen Yu, Hui Cui, Guo-Min Ji, Shu-Qi Li, Yong Huang, Ruo-Ning Wang, Wen-Feng Xiao

**Affiliations:** 1 Magnetic Resonance Room, Ji Nan Zhang Qiu District hospital of TCM, Shan Dong, 250200 China; 2 CT Diagnosis Room, Liao Cheng People’s hospital, Shan Dong, 252000 China; 3 Department of Radiology, Gu Cheng People’s hospital, He Bei, 053000 China; 4 Department of Ultrasonics, Lan Ling People’s hospital, Shan Dong, 277799 China; 5 Department of Radiology, Shandong Tumor hospital, Ji Nan, 250117 China; 6 Minimally Invasive Center, Tumor hospital, Sun Yat-Sen University, Guangzhou, 510060 China; 7 Magnetic Resonance Room, Sheng Li Oilfield Central Hospital, Shan Dong, 257034 China

**Keywords:** Heart, Angiosarcoma, Computed tomography

## Abstract

**Background:**

To investigate the CT manifestations of primary cardiac angiosarcoma.

**Methods:**

The clinical and CT data for 9 patients with cardiac angiosarcoma were retrospectively analyzed.

**Results:**

The lesions in all nine cases were located in the right atrium. In two cases, the involved lesion led downward to the tricuspid valve and right ventricle, and the dynamic cine showed that the lesion affected the opening and closing of the tricuspid valve. In three cases, the lesion involvement led to a thickened pericardium, accompanied by pericardial effusions. On CT plain scans, six patients showed homogeneous density, while three showed inhomogeneous density, two of which were associated with bleeding. On enhanced CT scans, seven patients showed heterogeneous centripetal enhancement, and some angiograms showed lesions with tortuous small blood vessels. The remaining two cases showed early stage rapid inhomogeneous enhancement. Five cases showed multiple metastatic nodules in the lungs at the time of initial diagnosis; four of these showed distinct sharp edges in multiple pulmonary nodules.

**Conclusions:**

Cardiac angiosarcoma has a predilection site and is prone to invading adjacent structures, manifesting as malignant pericardial and pleural effusions. The CT enhancement manifestations are mostly inhomogeneous and centripetal with ground-glass opacity peripheral to the intrapulmonary metastases.

## Background

Primary cardiac angiosarcoma (AS) is a clinically rare (incidence of approximately 0.017%) and highly invasive cardiac tumor with a poor prognosis [1, 2], the overall prognosis of patients with primary cardiac sarcoma is poor, with median overall survival (OS) ranging from 9 to 27 months in recent case series [3], which has previously been reported on a mostly case-by-case basis. Herein, we report the clinical and CT data for nine cases of primary cardiac AS that were collected from two hospitals over 10 years to improve understanding the disease’s characteristics at the cardiac site.

## Methods

### Clinical data

Nine cases of cardiac AS that had CT data and were pathologically confirmed from May 2007 to January 2018 were included in this study. Among the nine patients were 5 males and 4 females, ranging in age from 25 to 73 years, with an average age of 48.9 years; their disease histories ranged from 2 weeks to over 19 months. Clinical symptoms included chest pain, chest tightness, vomiting, cough, hemoptysis, shortness of breath, and fatigue. No patients had histories of drug abuse, special family histories, or jaundice at the time of the physical examination. From the laboratory examinations, seven cases showed abnormalities on the 8-item blood coagulation test (mainly prolonged prothrombin time and an increased prothrombin time ratio).

### CT examination

Nine cases were performed with CT plain scan + enhanced scan, and the scan parameters were as follows:

1. Six patients were subjected to scanning via the Philips Brilliance 64-slice CT system, coupled with the Extended Brilliance Workspace post-processing workstation: tube voltage - 140 kV; tube current - 300 mA (with dynamic current regulation); collimator combination - 64 × 0.625 mm; pitch - 0.891; reconstruction matrix - 512 × 512; scan field - 350 cm; and reconstruction slice thickness - 1 mm.
2. Three patients were subjected to the plain scan and enhanced scan using the Philips 256-slice Brilliance iCT system from the thoracic inlet to 2 cm below the diaphragm: tube voltage - 120 kV; tube current - 300 mA (with dynamic current regulation); collimator combination - 64 × 0.625 mm; scan slice thickness - 5 mm; and reconstruction slice thickness - 1.25 mm.

Eighty milliliters of non-ionic contrast agent (iodine, 370 mg/mL) + 30 mL normal saline was intravenously injected through the cubital vein at a rate of 3.5 mL/s using a high-pressure syringe. Arterial phase scanning was performed after a 20-s delay, venous phase scanning was performed after a 55- to 60-s delay, and delayed phase scanning was performed after a 150-s delay.

### Image analysis

Two radiologists (JFY,HC), both with more than 15 years of experience in diagnostic imaging of the cardiovascular system, analyzed the CT manifestations for all cases, especially the lesion growth site, internal components, infiltration range, and enhancement characteristics.

### Pathological analysis

Specimens from all cases were paraffin-embedded, sectioned, stained with hematoxylin and eosin (HE), and immunohistochemically typed. Three radiologists with more than 10 years of experience in pathological diagnoses read the images.

## Results

### General results and follow-up

Five patients received no surgical treatment due to extensive metastases. Of these, three underwent chemotherapy and died 9–17 months after diagnosis; the other two were not treated at this hospital and were lost to follow-up.

Two patients with small tumors had radical resection. Two patients had a thoracotomic biopsy only because surgery showed that the tumor had invaded too extensively. Postoperative follow-up: One patient relapsed 7 months post-surgery, developing lung, liver and spleen metastases. The disease condition progressed after 4 courses of chemotherapy, and the patient died 13 months post-operation. Three patients received no further treatment, and all died within 9 months post-surgery. One patient survived less than 2 months post-surgery. No cases showed long-term survival.

### CT results

**Fig. 1:**
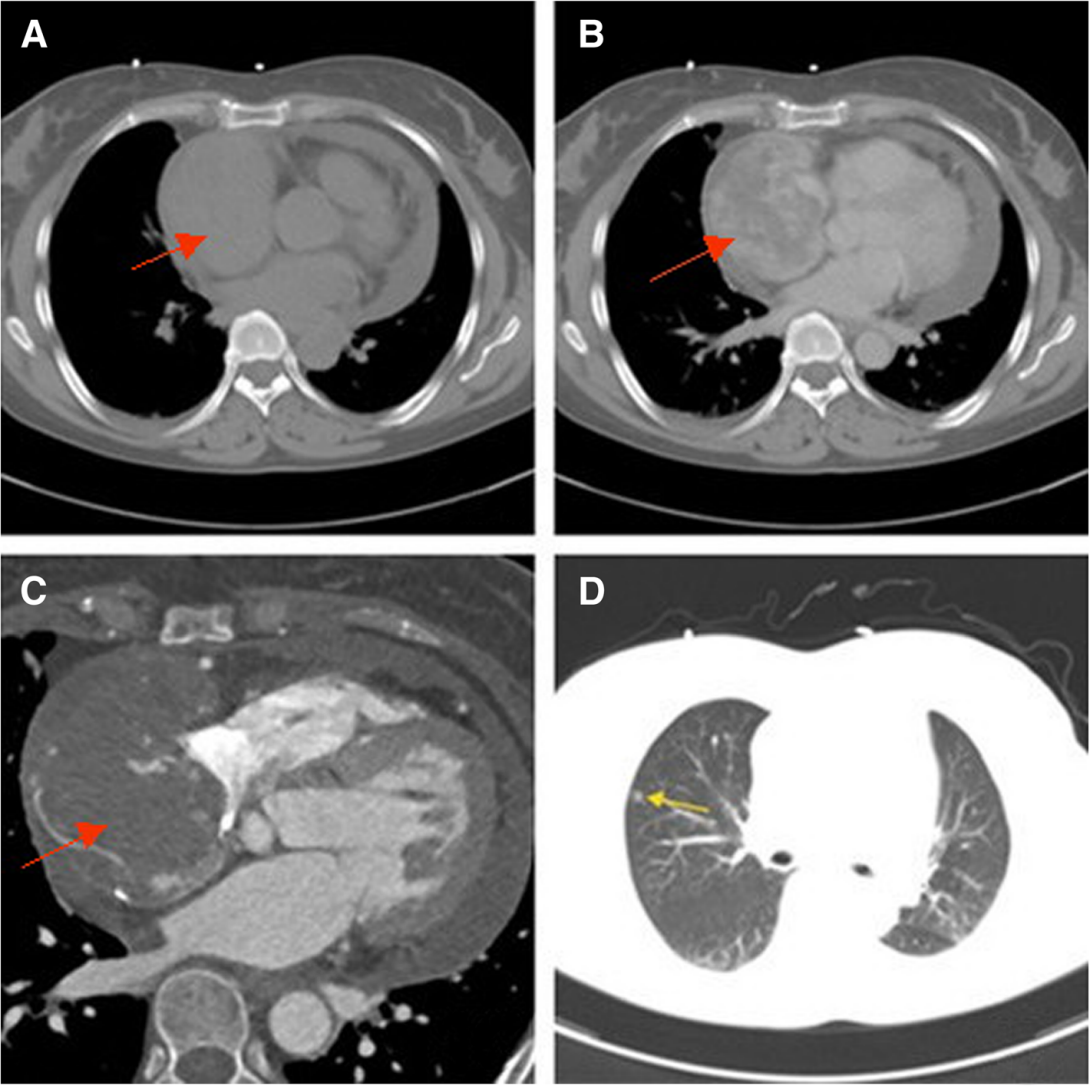
Female, 56 years old, right atrium angiosarcoma (red arrows). **a**: Plain CT scan shows an equal-density mass in the right atrium. The density is partly homogenous, and pericardial and pleural effusion are noted. **b**, **c**: Enhanced CT scan indicates inhomogeneous mass enhancement and an angiogram of tortuous small blood vessels. **d**: Metastatic nodules (yellow arrows) are seen in the right lung

**Fig. 2:**
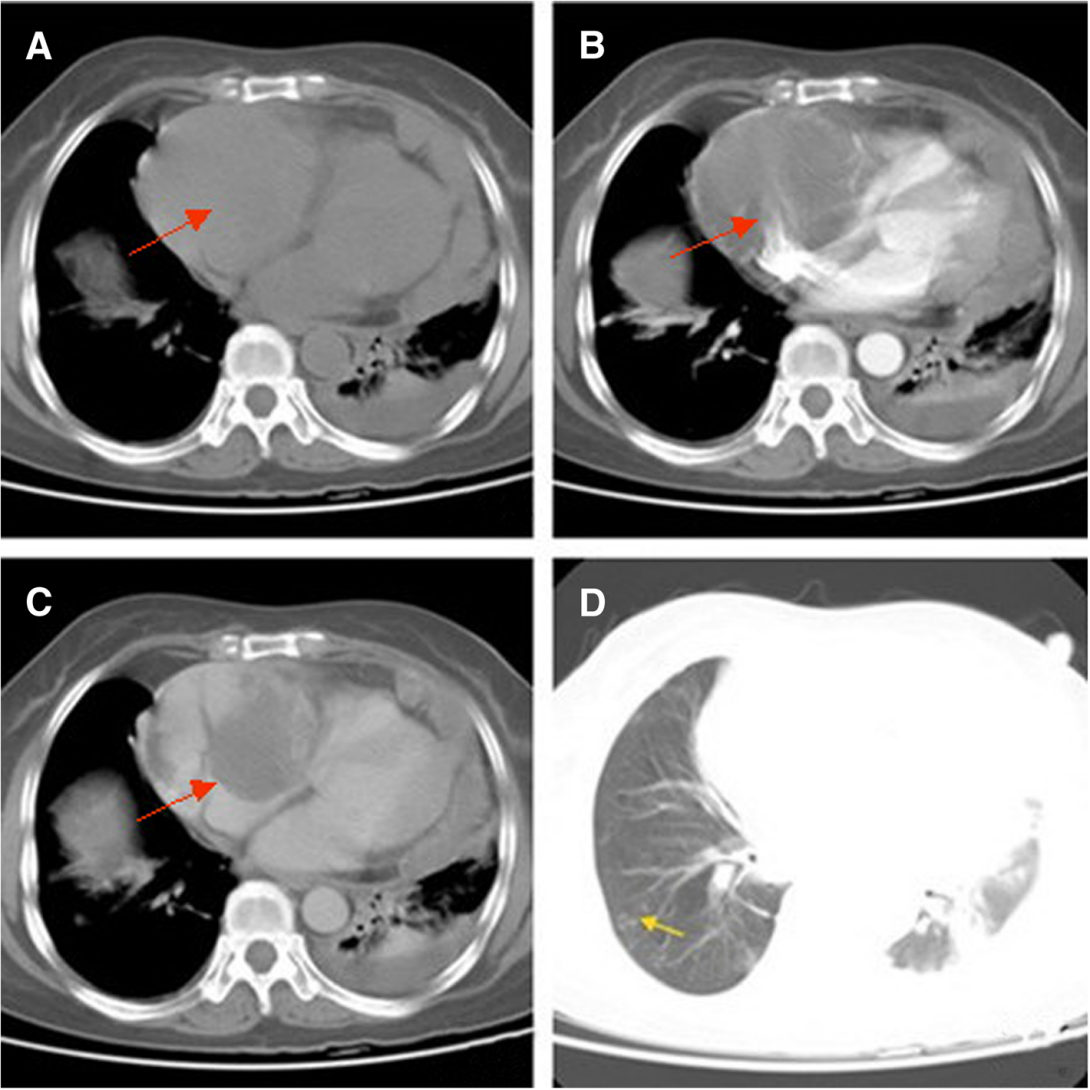
Female, 53 years old, right atrium angiosarcoma (red arrows). **a**: Plain CT scan shows an equal-density mass in the right atrium. The density is partly homogenous, and pericardial and pleural effusion are noted. **b**, **c**: Enhanced CT scan indicates inhomogeneous and centripetal mass enhancement, and multiple nodules are seen on the pericardium. **d**: Metastatic nodules (yellow arrows) are seen in the right lung

Lesions in all nine cases ranged from 4.3 cm to 10.9 cm in diameter, with an average diameter of 5.8 cm. The main tumor masses were all located at the right atrium, leading to significantly increased atrial size. In one case, the lesion involved the inlet of the superior vena cava, leading to filling defects in the superior vena cava. In two cases, the involved lesion led downward to the tricuspid valve and right ventricle, and the dynamic cine showed that the lesion affected the opening and closing of the tricuspid valve. In three cases, the pericardium was thickened with pericardial effusions. Of these, two were associated with pleural effusion (Figs. 1a and 2a). On CT plain scans, six patients showed homogeneous density, and three showed inhomogeneous density, two of which were associated with bleeding. On enhanced CT scans, seven patients showed heterogeneous centripetal enhancement (Figs. 1b and 2b, c) and marginal enhancement in the arterial phase. The enhancement scope extended to the center of the lesion in the delayed phase, in which the enhancement degree was more pronounced than that in the arterial phase. Some lesions showed angiograms of tortuous small blood vessels (Fig. 1c). The remaining two cases showed early stage rapid inhomogeneous enhancement.

**Fig. 3:**
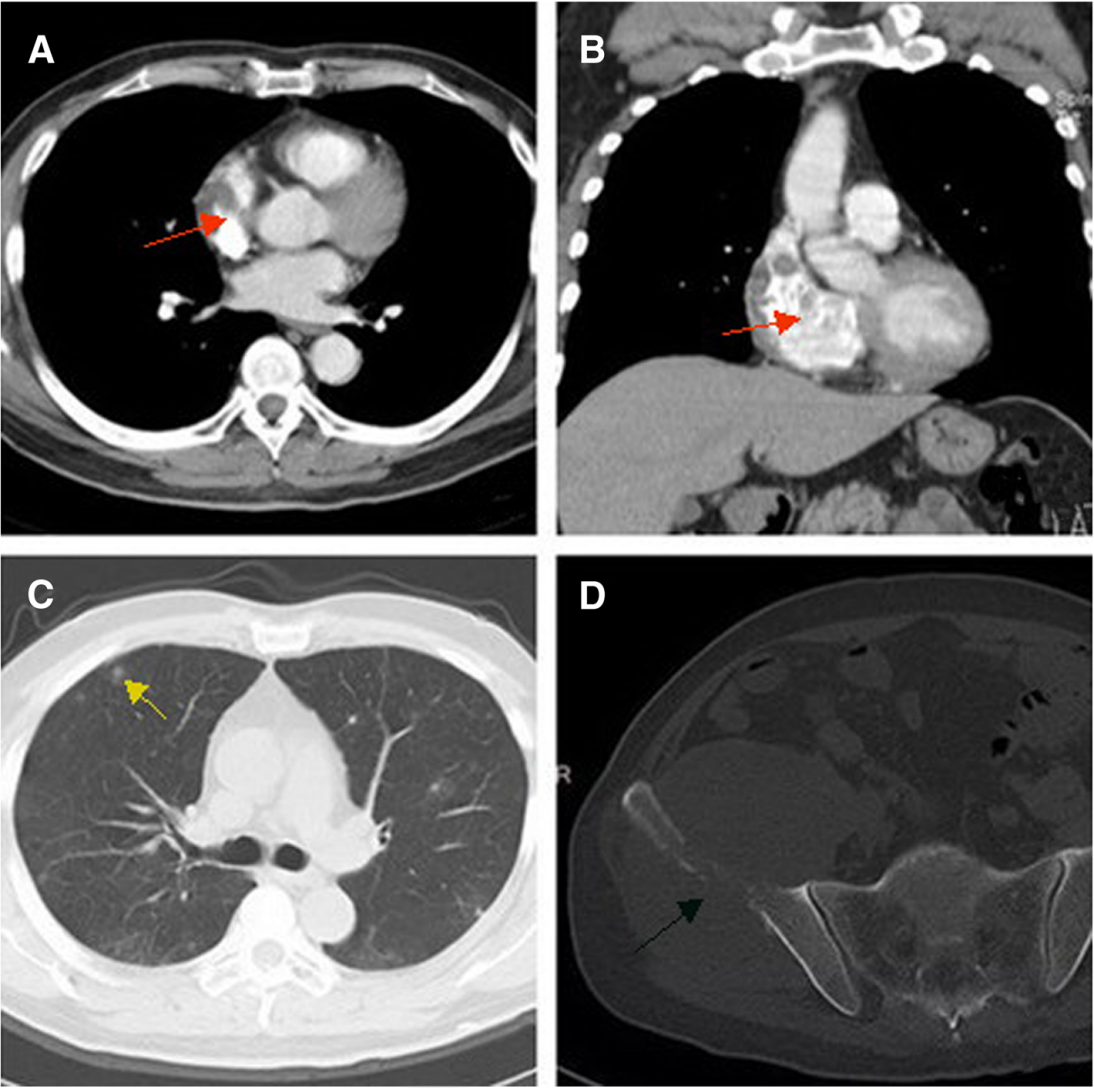
Male, 43 years old, right atrium angiosarcoma. **a**, **b**: Enhanced CT scans on both transverse sections and coronal sections indicate a mass with inhomogeneous enhancement (red arrows); **c**: Multiple metastatic nodules are seen in the bilateral lungs (yellow arrows), with ground-glass opacity in the nodule peripheries. **d**: The right ilium is involved (balck arrows), exhibiting osteolytic bone destruction, a blurry boundary, and a massive soft tissue mass

In five cases, multiple metastatic nodules were observed in the lungs upon initial diagnosis, of which four showed distinct sharp edges in multiple pulmonary nodules (Figs. 1d and 2d), while one showed ground-glass opacity at the pulmonary nodule peripheries (Fig. 3c). Two cases showed complications of iliac involvement upon initial diagnosis (Fig. 3d), in which the ilium manifested osteolytic bone destruction with slight expansion and indistinct boundaries and the lesions perforated the bone cortices to form soft tissue masses. In two cases, spleen complications and liver metastases were noted, and the mass enhancement was similar to that of cardiac AS.

### Microscopic observation results

Tumor cells of the right atrial mass were aligned in a solid and papillary manner and locally formed vascular cavity-like structures. The cavities were irregular and connected, appearing as holes and fissures. The tumor grew invasively, showing thrombus locally. Immunohistochemistry results were as follows: CD34+, CD31+, Fli-1+, CD117+, SMA-, Desmin-, S-100-, CKpan-, and Ki-67 + .

## Discussion

AS is rare, accounting for approximately 1% of all sarcomas [4]. AS most frequently occurs in the head, neck, and breast. Heart tumors are rare and most are benign, especially in cardiac myxomas. AS is one of the most common primary cardiac malignancies, accounting for approximately 40% of all cases, followed by rhabdomyosarcoma [5]. Primary cardiac AS is the most aggressive cardiac tumor and can occur at any age but is more common in those 30–40 years of age. Primary cardiac AS can occur anywhere in the heart, but most tumors occur on the right side, especially the right atrium, and rarely in the epicardium, pericardium, and right ventricle [6]. In most cases, cardiac AS metastasizes to the lungs and bone, occasionally to the liver and spleen, and extremely rarely to the brain [7]. Primary cardiac AS usually causes chest pain, vomiting, cough, hemoptysis, shortness of breath, fatigue, and arrhythmia [8]. One patient in this group had arrhythmia 3 years prior and had been treated with radiofrequency ablation. Upon recent diagnosis, this patient was found with multiple metastatic lesions in the lungs, liver and spleen. Cardiac AS has a poor prognosis, with a median survival of 5 to 13 months. The poor prognosis is caused by several factors including high tumor invasiveness, difficulty in having a complete resection due to the unique surgical site, poor response to adjuvant therapy, and a lack of targeted treatment [7, 9, 10].

In most cases, cardiac AS etiology remains unclear [11]. Occasionally, patients with cardiac AS had ionizing radiation exposure and long-term exposure to chemically synthesized materials. At present, cardiac AS is treated mainly by surgical resection combined with radiotherapy and chemotherapy [12, 13]. Two patients in this group received right atrial mass resection + right atrial reconstruction and tricuspid annuloplasty depending on the scope of the infiltration. In other cases, the lesions were too large and had already invaded the pericardium, right ventricle and undergone distant metastases; thus, surgery was not performed, and radiotherapy and chemotherapy were conducted to reduce the mass. However, high-dose radiotherapy and anthracycline-based chemotherapy are cardiotoxic, making it controversial to perform this combination on patients with advanced AS. Paclitaxel has been used as a radiosensitizer to reduce the adverse effects of high-dose radiation therapy [14]. Carboplatin and paclitaxel with radiotherapy have also achieved good therapeutic effects [15].

Under plain CT scanning, similar to AS in other sites such as the liver and spleen, cardiac AS shows homogeneous or inhomogeneous density, sometimes with hemorrhaging. Under enhanced scanning, most lesions show inhomogeneous centripetal enhancement without homogeneous enhancement, and complete centripetal filling is seen in benign AS cases. AS tissue shows a discontinuous vascular network microscopically, forming no new blood vessels in some areas, and only single tumor cells are seen floating in a blood pool, making it difficult for the contrast agent to penetrate the entire lesion. Conversely, AS tumor cells are well-differentiated, with a completely connected neovascular network, making it possible for the contrast agent to slowly and continuously spread from the edge to the center of the tumor. Cardiac computed tomography (CT), cardiac MRI, and PET/CT can help determining the extent of infiltration and the presence of potential metastases. Cardiac MRI enables better soft-tissue and tumor characterization, and it is superior in revealing tumorous infiltration of the myocardium and pericardium [16]. Cardiac angiosarcoma is mostly an intramural neoplasm infiltrating the wall and the pericardium and protruding into the right cardiac cavities infiltrating the inferior vena cava and the tricuspid valve [17–19]. Cardiac AS is typically accompanied with multiple lung metastatic nodules. Five patients (5/9) developed lung metastases, of which, one showed ground-glass opacity in the nodule peripheries, but the cause of this remains unknown (due to the reduced gas retention in the peripheral alveoli or thickened alveolar walls).

## Conclusion

Cardiac AS usually occurs in the right atrium and can invade the pericardium, right ventricle, superior vena cava, and tricuspid valve. Cardiac AS can cause malignant pericardial and pleural effusions, manifesting a series of clinical symptoms. Enhanced CT imaging of cardiac AS is consistent with the inhomogeneous centripetal enhancement that is typical to AS, and ground-glass opacity peripheral to the intrapulmonary metastasis is common, which can indicate the disease. Due to its unique disease site, strong invasiveness, late detection, surgical difficulty and incomplete surgical resection, cardiac AS patients usually have short median survivals and poor prognoses.
